# Supplementary material for: Co-production of an online research and resource platform for improving the health of young people—The hype project
Source: PLoS One. 2024 Jun 7;19(6):e0277734. doi: 10.1371/journal.pone.0277734 (PMC11161015; doi:10.1371/journal.pone.0277734)
Supplement: Attachment — Submitted filename: Response to Reviewers.pdf [file pone.0277734.s001.pdf]

Dear Professor Apurva Kumar Pandya,

Thank you for your letter requesting that we revise and resubmit our manuscript entitled " Co-production of an online research and resource platform for improving the health of young people - The HYPE Project." recently submitted to the PLOS ONE (PONE-D-22-29685).

We are grateful to your reviewers for their helpful suggestions. In the response to reviewers' document, we have included a reply to their comments and explain how we have revised the paper (below in bold italics). We have provided both clean and highlighted (indicating revisions) versions of our paper as part of our re-submission.

We hope our responses and our revised article are satisfactory.

Thank you in advance for re-considering this manuscript.

Sincerely,
Dr Cerisse Gunasinghe

Authors' response

Revisions relating to journal requirements:

1. Please ensure that your manuscript meets PLOS ONE's style requirements, including those for file naming.

Apologies for not adhering to the guidelines set by PLOS ONE. After careful consideration and review of the PLOS ONE guidelines, we have made the necessary revisions to ensure that our manuscript aligns with the journal's requirements. Specifically:

The title page has been amended in line with the template found at https://journals.plos.org/plosone/s/file?id=ba62/PLOSOne_formatting_sample_title_authors_affiliations.pdf

The main text has been amended in line with the template found at https://journals.plos.org/plosone/s/file?id=wjVg/PLOSOne_formatting_sample_main_body.pdf

The files have also been renamed as follows

- **Response to Reviewers**
- **Revised Manuscript with Track Changes**
- **Manuscript**

2. You indicated that you had ethical approval for your study. In your Methods section, please ensure you have also stated whether you obtained consent from parents or guardians of the minors included in the study or whether the research ethics committee or IRB specifically waived the need for their consent.

We have added to the Methods and Material section the following statement:

"The research ethics committee approved consenting processes for individuals 16-18 years (with capacity to consent). Those who did not have capacity to consent were not able to provide data for research purposes."

3. Thank you for stating in your Funding Statement:

"This paper represents independent research funded by the NIHR Maudsley Biomedical Research Centre at South London and Maudsley NHS Foundation Trust and King's College London. The views expressed are those of the author(s) and not necessarily those of the NHS, the NIHR or the Department of Health and Social Care. SH is part-funded by the NIHR Maudsley Biomedical Research Centre at South London and Maudsley NHS Foundation Trust and supported by the Economic and Social Research Council (ESRC) Centre for Society and Mental Health at King's College London (ESRC Reference: ES/S012567/1). SH also receives funding from Impact on Urban Health and Wellcome Trust. C.W. is supported by the ESRC Centre for Society and Mental Health at King's College London (ESRC Reference: ES/S012567/1). The funders did not have a role in the study design; collection, analysis or interpretation of data; the writing of the manuscript; or in the decision to submit the manuscript for publication. These data can be accessed through the corresponding author."

Please provide an amended statement that declares *all* the funding or sources of support (whether external or internal to your organization) received during this study, as detailed online in our guide for authors at http://journals.plos.org/plosone/s/submit-now. Please also include the statement "There was no additional external funding received for this study." in your updated Funding Statement.

Please include your amended Funding Statement within your cover letter. We will change the online submission form on your behalf.

We have amended the Funding Statement as follows:

"This paper represents independent research funded by the National Institute of Health Research (NIHR) Maudsley Biomedical Research Centre at South London and Maudsley NHS Foundation Trust and King's College London (Grant number BRC-1215–20018). The views expressed are those of the author(s) and not necessarily those of the National Health Service, the NIHR or the Department of Health and Social Care. CG and NB are fully funded by the NIHR Maudsley Biomedical Research Centre at South London and Maudsley NHS Foundation Trust (Grant number BRC-1215–20018). SLH was part-funded by the NIHR Maudsley Biomedical Research Centre at South London and Maudsley NHS Foundation Trust (Grant number BRC-1215–20018) and is supported by the Economic and Social Research Council (ESRC) Centre for Society and Mental Health at King's College London (Grant Reference: ES/S012567/1). SLH also received funding from the Wellcome Trust (Grant Reference: 203380/Z/16/Z) and Impact on Urban Health part of Guy's & St Thomas' Foundation [Grant References: EIC210605 and EIC221208). CW is supported by the ESRC Centre for Society and Mental Health at King's College London (Grant Reference: ES/S012567/1). There was no additional external funding received for this study. The funders had no role in study design, data collection and analysis, decision to publish, or preparation of the manuscript."

We also request that the following statement also be included as required by our funder.

The views expressed are those of the author(s) and not necessarily those of the NHS, the NIHR or the Department of Health and Social Care.

4. We **note** that Figure 2 in your submission contain [map/satellite] images which may be copyrighted. All PLOS content is published under the Creative Commons Attribution License (CC BY 4.0), which means that the manuscript, images, and Supporting Information files will be freely available online, and any third party is permitted to access, download, copy, distribute, and use these materials in any way, even commercially, with proper attribution. For these reasons, we cannot publish previously copyrighted maps or satellite images created using proprietary data, such as Google software (Google Maps, Street View, and Earth). For more information, see our copyright guidelines: http://journals.plos.org/plosone/s/licenses-and-copyright.

We require you to either (1) present written permission from the copyright holder to publish these figures specifically under the CC BY 4.0 license, or (2) remove the figures from your submission

We have removed this figure from our submission and manuscript.

Revisions relating to reviewers' comments:

Reviewer #1:

INTRODUCTION
The introduction is written well in terms of outlining the need for the HYPE platform, however the paper is much more about the coproduction of the platform through advisory groups and stakeholder engagement and the online nature of the intervention. It would better situate the reader if more evidence was presented at this stage about the different online platforms that are out there for young people, how they have used co-production in their development (or not) and more about 'the work of van Germert-Pijnen and the proposed five key aspects in optimising the engagement of the target population in online/web-based health interventions and resource. For example we know that TikTok is being used as a search engine by young people. I feel the introduction would benefit from focusing on the way young people engage online. Also you mention that the work was guided by Germert-Pijnen and Levesque, however this is not revisited in the analysis or the discussion. How did these inform the study?
I suggest removing the qualitative study questions and hypothesis as this confuses the purpose of the paper. This whole section created confusion as to what you were presenting.

We have amended the Introduction to incorporate more about, the different online platforms that are available for young people; the work of van Germert-Pijnen and removed the research questions and hypotheses. Also please see below regarding revisions to the Analysis and Discussion that address the points made in relation to clarifying how existing models by van Germert-Pijnen and Levesque informed this programme of work.

METHODS:
It is clear that there was lots of engagement and work gone into making the platform relevant for young people which is admirable and well thought through, it is a great programme. However, the section between the introduction and the results is convoluted and would benefit from being considerably reduced with better use of figures/table/graphics to demonstrate many of the steps. I suggest the following:
• A creative figure showing the different elements of the HYPE programme and which are being presented in this paper as I struggled to keep focus on your main aim.
• A diagram or table showing the stages of research, the methods and the number of participants that took part in the different stages. For example, I could not decipher how many people took part in FDGs, how many took place and how often.
• Cut the level of detail provided on the process and just focus on giving the reader an overview of

what you did
• A section that describes the membership of the advisory group, including diversity and numbers, drop out rates etc, to give a feel for who was advising the development of the project and how they found it. Also as the title has the word co-production in it, I would expect to see some reflexivity and consideration of power. Are any of the authors from the advisory group? I would expect their input on this if it is really adhering to co-production principles.
Line 63 sentence needs 'this' removing. 'Integrated health and social care for this young people (32) that is age and culturally appropriate (32, 33) is critically needed to improve mental health outcomes.
256 the HYPE project advisory group members provided feedback and made 257 suggestions about intended project procedures.- how many did this?
Line 357 This is quite a big statement, what is it based on - It is estimated that these rates would be doubled or more if data were available for those under 18 years.
Limitation google analytics for under 16s – is this in limitations?

Thank you for these helpful recommendations which we consider will help improve the presentation of the engagement and research activities associated with the HYPE project. The Methods and Materials section has been significantly revised. We have added an additional diagrams:

1. **A figure showing the different elements of the HYPE programme and which are being presented in this paper focusing on our main aim (see Figure 1).**
2. **A diagram showing the stages of research, the methods and the number of participants that took part in the different stages (see Figure 3)**

Within the section "Establishing the HYPE stakeholder and advisory groups", lines 151-154 (clean version of manuscript) has been edited to describe the membership of the advisory group, including their diversity, "In addition, the HYPE project advisory group was formed which included co-authors (CG, NB, SM, RR, CW, JJN & SLH), the HYPE project research team, school/college and undergraduate students, health service users and young people who had previously attended HERON engagement and outreach activities. This advisory group was diverse in relation to age, gender, ethnicity, and race." We have added a diagram (Figure 2) to illustrate information about how many participants took part in each stage of the project development and implementation as well as clarification of drop-out rates within the text of the manuscript. We have also added a section on reflexivity and made more explicit the co-authors involvement in the advisory groups.

We have removed 'this' from the sentence 'Integrated health and social care for this young people (32) that is age and culturally appropriate (32, 33) is critically needed to improve mental health outcomes.'

We hope that the provision of and reference to Figure 3 addresses your question of how many HYPE project advisory group members provided feedback and made suggestions about intended project procedures.

We have removed the line "It is estimated that these rates would be doubled or more if data were available for those under 18 years."

We have added the inability to use google analytics for under 16s in the methodological considerations section.

RESULTS

The results section is very short and I am not sure it delivers what you intend. I felt that some of the results were interspersed in the methods and discussion sections. Also I couldn't find the results from the FDGs or any themes that came out of the thematic analysis. Was it the resources section? This needs much more clarity. Again suggest being clear what methods and data you are presenting and ensure that it links to your key messages and aims.

After some consideration, we have removed the results from the focus group discussions to help focus the paper on the two main aims of the paper - to describe the development and implementation of the HYPE project platform and to describe the characteristics of participants who engaged with this type of online pilot research survey and associated resource platform. We have amended the methods, results and discussion section for further clarification.

DISCUSSION

The discussion feels like an extension of the results, suggest better links to the theory and concepts earlier in the introduction and presenting some key take home messages that draw and situate the study in the current literature.

This section has been revised to address the points made above and the highlight key messages.

Overall, we have narrowed the focus of the paper as recommended by reviewer 1 and hope this improves the flow for readers yet still offers transparency and sufficient detail should those working with the study population in future, be able to replicate the methodology presented.
